# Second- and third-line systemic therapy in patients with advanced esophagogastric cancer: a systematic review of the literature

**DOI:** 10.1007/s10555-016-9632-2

**Published:** 2016-07-14

**Authors:** Emil ter Veer, Nadia Haj Mohammad, Gert van Valkenhoef, Lok Lam Ngai, Rosa M. A. Mali, Martijn G. H. van Oijen, Hanneke W. M. van Laarhoven

**Affiliations:** 1 Department of Medical Oncology, Academic Medical Centre, University of Amsterdam, Amsterdam, The Netherlands; 2 Department of Epidemiology, University of Groningen, University Medical Centre Groningen, Groningen, The Netherlands

**Keywords:** Advanced esophagogastric cancer, Chemotherapy, Targeted therapy, Second-line, Third-line, Meta-analysis

## Abstract

The optimal second- and third-line chemotherapy and targeted therapy for patients with advanced esophagogastric cancer is still a matter of debate. Therefore, a literature search was carried out in Medline, EMBASE, CENTRAL, and oncology conferences until January 2016 for randomized controlled trials that compared second- or third-line therapy. We included 28 studies with 4810 patients. Second-line, single-agent taxane/irinotecan showed increased survival compared to best supportive care (BSC) (hazard ratio 0.65, 95 % confidence interval 0.53–0.79). Median survival gain ranged from 1.4 to 2.7 months among individual studies. Taxane- and irinotecan-based regimens showed equal survival benefit. Doublet chemotherapy taxane/irinotecan plus platinum and fluoropyrimidine was not different in survival, but showed increased toxicity *vs*. taxane/irinotecan monotherapy. Compared to BSC, second-line ramucirumab and second- or third-line everolimus and regorafenib showed limited median survival gain ranging from 1.1 to 1.4 months, and progression-free survival gain, ranging from 0.3 to 1.6 months. Third- or later-line apatinib showed increased survival benefit over BSC (HR 0.50, 0.32–0.79). Median survival gain ranged from 1.8 to 2.3 months. Compared to taxane-alone, survival was superior for second-line ramucirumab plus taxane (HR 0.81, 0.68–0.96), and olaparib plus taxane (HR 0.56, 0.35–0.87), with median survival gains of 2.2 and 4.8 months respectively. Targeted agents, either in monotherapy or combined with chemotherapy showed increased toxicity compared to BSC and chemotherapy-alone. This review indicates that, given the survival benefit in a phase III study setting, ramucirumab plus taxane is the preferred second-line treatment. Taxane or irinotecan monotherapy are alternatives, although the absolute survival benefit was limited. In third-line setting, apatinib monotherapy is preferred.

## Introduction

Worldwide, advanced esophageal and gastric cancers are major causes of mortality [1]. In the first-line setting, fluoropyrimidine and platinum combinations are preferred [2]. As virtually all patients become resistant to first-line treatment, effective second- or later-line treatments are warranted. Previously, it has been shown that single-agent irinotecan and taxane as second-line chemotherapy increase survival compared to best supportive care (BSC) [3, 4]. Also, targeted agents that were shown to be active in clinical trials have been introduced into clinical practice, for example ramucirumab, a vascular endothelial growth factor receptor-2 (VEGFR-2) inhibitor [5, 6]. Although evidence for active treatments after progression on first-line (chemo)therapy has been established, to date, there are several questions that remain unanswered.

First, as “salvage” chemotherapy usually consists of irinotecan or taxane, these two strategies are generally regarded as equally effective [7]. However, a literature review to assess the possible differences in efficacy, defined as the maximum effect achievable for a drug in clinical trial setting, and safety of irinotecan and taxane is not available. Second, to increase the efficacy of second-line irinotecan or taxane single-agent chemotherapy, several trials have been conducted in which another cytotoxic agent, for example platinum or fluoropyrimidine, was added to a backbone of irinotecan or taxane. However, the results of these randomized controlled trials (RCT) are inconsistent and despite the publication of a recent systematic review [8], doublet chemotherapy compared to single chemotherapy including the newest RCTs has not been investigated in a fluoropyrimidine add-on and platinum add-on subgroup structured meta-analysis yet. Third, safety data were not included in recent reviews or meta-analyses, which makes it more difficult to put the findings into a clinical perspective [3, 4, 9]. Fourth, many small trials have been conducted with targeted agents that did not receive much attention in literature reviews or meta-analyses [10, 11] since usually only larger phase III trials have been included [5, 6, 12]. Overview of the smaller trials will help to identify the potentially most efficacious targeted agents for future studies. Fifth, in addition to second-line therapy, also third- or later-line therapy has been subject of investigation lately, but an overview is currently missing [13, 14]. Finally, usually only relative effect sizes are used in meta-analysis, which may be difficult to interpret in clinical practice. In order to enhance the clinical applicability of the findings, also a more absolute efficacy summary statistic should be incorporated into literature reviews or meta-analyses, for example the absolute median survival gain from an experimental treatment over the control treatment/best supportive care.

In sum, the evidence regarding all possible second- or third-line treatments is inadequately summarized, which may be difficult for decision-making in clinical practice. Therefore, we conducted a systematic review and meta-analysis of all currently available randomized controlled trials (RCTs).

## Methods

### Literature search

Medline, EMBASE and the Cochrane Central Register of Controlled Trials (CENTRAL) were searched for eligible RCTs up to January 2016. The search strategy consisted of medical subject headings (MeSH) combined with text words for esophageal and gastric cancer and with text words associated with second- or later-line therapy (Table 1). Also, the conference abstracts of the American Society of Clinical Oncology (ASCO) and European Society for Medical Oncology (ESMO) between 1990 and January 2016 were searched. NHM and EtV screened the titles, abstracts, and full texts independently. Disagreements were discussed with a third arbiter (HvL) until consensus was reached.

**Table 1:** Full search strategy

Medline via Pubmed
(“stomach neoplasms” [MeSH terms] or “esophageal neoplasms” [MeSH terms]) and (((refractory [title/abstract] or previously treated [title/abstract]) or salvage treatment [title/abstract]) or second line [title/abstract]) and Clinical trial [ptyp]
EMBASE via Ovid
1. esophagus tumor/or exp esophagus cancer
2. stomach tumor/or exp stomach cancer
3. ((esophag* or oesophag* or stomach or gastric or gastroesophag* or gastrooesophag*)adj5 (neoplas* or cancer* or carcino* or adenocarcino* or tumor or tumors or tumour or tumours or malig*)).ti,ab.
4. refractory.mp.
5. previously treated.mp
6. salvage treatment.mp.
7. second line.mp.
8. 1 or 2 or 3
9. 4 or 5 or 6 or 7
10. exp controlled clinical trial/or randomized.ti,ab. or randomised.ti,ab. or placebo.ti,ab. or randomly.ti,ab. or trial.ti
11. 8 and 9 and 10
Additional filters:
1. year = “2005–2016”
2. not (conference abstract or conference paper or “conference review” or conference proceeding)
3. articles
Central Register of Controlled Trials (CENTRAL)
#1 MeSH descriptor: [esophageal neoplasms] explode all trees
#2 MeSH descriptor: [stomach neoplasms] explode all trees
#3 #1 or #2
#4 (refractory): ti,ab,kw
#5 (previously treated): ti,ab,kw
#6 (salvage treatment): ti,ab,kw
#7 (second line): ti,ab,kw
#8 #4 or #5 or #6 or #7
#9 #3 and #9
Additional filter: trials
Conference search
Searching journal content for *gastric* (all words) in title or abstract and random* or advance* OR metasta* (all words) in full text, from Jan 2004 through Jan 2016 in http://www.ascopubs.org/search and http://www.annonc.oxfordjournals.org/search

### Study selection

Studies had to meet the following criteria of eligibility: (1) prospective phase II or III randomized controlled trials; (2) included patients with pathologically proven metastatic, unresectable, or recurrent adenocarcinoma of the esophagus, gastro-esophageal junction (GEJ), or stomach; (3) patients were previously treated with systemic therapy.

### Data extraction and quality assessment

The major efficacy outcome of interest was overall survival (OS), since an international expert consensus panel stated that OS as endpoint in oncology clinical trials is most appropriate [15]. Other outcomes of interest were progression-free survival (PFS) and the incidence of grade 3–4 adverse events (AEs) to assess the safety (http://ctep.cancer.gov). Two reviewers (LN and RM) were involved in data extraction; discrepancies were solved by discussion with an arbiter (EtV). The quality of the included studies was assessed using the Cochrane Risk of bias tool (version 5.1.0). Items were scored as low, high, or unknown risk of bias.

### Statistical analysis

For time-to-event outcomes OS and PFS, hazard ratios (HR) with 95 % confidence intervals (95 % CI), number of events or *p* values were extracted to calculate the logHR and standard error based on intention-to-treat study populations [16]. Also, medians were extracted to calculate the absolute median OS and PFS gain (Δmedian) in months from an experimental treatment over the control treatment arm. The Δmedians were shown for individual studies and the range of Δmedians for comparisons with multiple studies. For the comparison of grade 3–4 AEs between groups, the number of events and sample-sizes were used to calculate risk ratios (RR) and 95 % CI’s. Review Manager 5.3 was used for statistical analysis.

First, we examined the efficacy and safety of second-line chemotherapy compared to best supportive care (BSC). Second, we compared the efficacy and safety of irinotecan- and taxane-based chemotherapy regimens. Third, the efficacy and safety of combination chemotherapy compared to chemotherapy-alone was examined. Fourth, single targeted agents were compared to a reference arm of BSC. Fifth, the added value of targeted therapy to chemotherapy compared to chemotherapy-alone was examined. Finally, targeted agents for specific molecular subgroups were examined.

In case of statistical heterogeneity, as tested with the Cochran Q and quantified by the *I*^2^ index, baseline characteristics in the corresponding studies were explored and subsequent sensitivity analysis conducted by omitting the heterogeneous studies. All comparisons were tested at a significance level of *α* = 0.05.

## Results

### Description of the studies

**Fig. 1:**
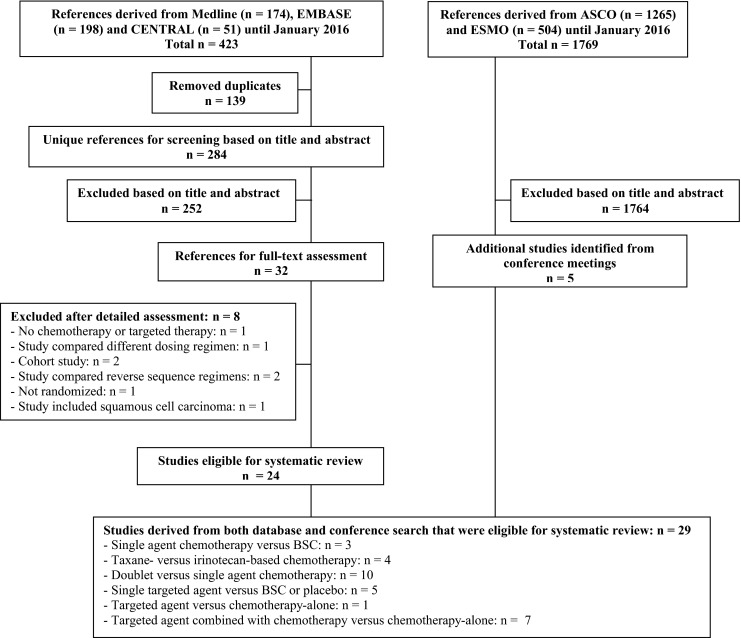
Flowchart of included studies. Flowchart of references derived from database search (*left*) and from conference search (*right*). Notes: the study of Kang and colleagues (2012) [19] was included in both the single-agent chemotherapy *vs*. BSC as well as the taxane- *vs*. irinotecan-based chemotherapy comparison

**Fig 2:**
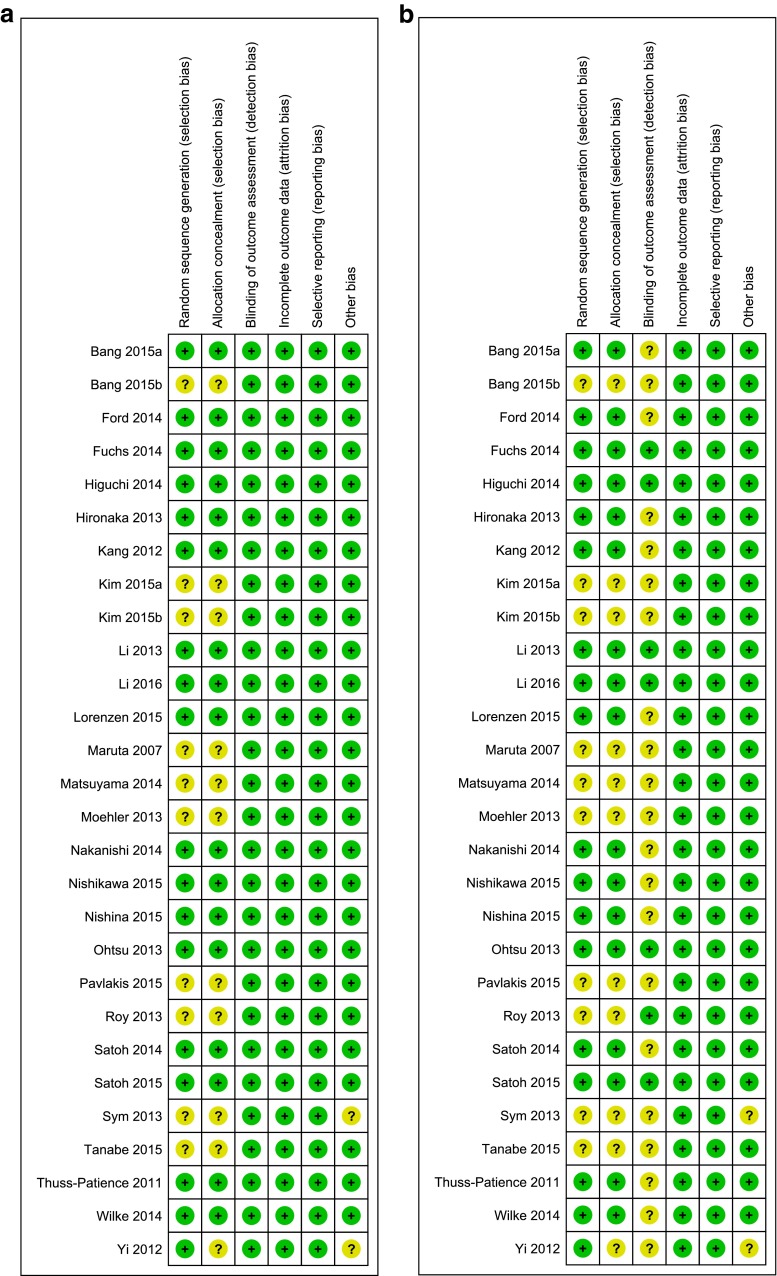
Risk of bias assessment for overall survival and progression-free survival. Risk of bias assessment for the primary outcome overall survival (**a**) and progression-free survival (**b**). The green spots with a “*plus sign*” indicate low risk of bias on an item, whereas the yellow spots with “*question mark*” indicate unknown risk of bias on an item. Notes: single-center studies and studies without a published full article report were rated unclear risk of other possible bias. The absence of a description of a blinded-imaging review committee was not regarded of bias for OS, since the primary outcome OS would not be influenced by this parameter

A total of 423 unique references were identified in Medline, EMBASE, and CENTRAL. Of the remaining 284 reports after title/abstract screening, 8 studies were excluded based on full text. Searching conference abstracts provided five additional studies. In total, 28 studies (*N* = 4810 patients) were included (Fig. 1). The number of studies that scored low risk of bias on all items of the Cochrane risk of bias tool for the primary outcome was 18 (64 %) (Fig. 2a). Five studies (18 %) were reported as meeting abstract or presentation. The risk of bias assessment for PFS is summarized in Fig. 2b. All patients included in the studies received a platinum and fluoropyrimidine-based first-line chemotherapy regimen (Table 2). No major differences in sex, age, disease status and Eastern Collaborative Oncology Group (ECOG) performance status were observed between the included studies, as shown in Table 2. In the majority of the studies, the inclusion was restricted to patients with an ECOG performance status of 0 or 1, as indicated in Table 2. In the following sections, recommendations about the performance status of patients to be eligible for a certain therapy are based on performance status as inclusion criterion of the specific trials (Table 2).

**Table 2:** Baseline characteristics

Study	*N*	Treatment arms	Sex male (%)	Age median (range)	Disease status metastatic (%)	ECOG PS inclusion	ECOG PS distribution		Treatment line	Prior treatment	Primary endpoint
							0–1 (%)	2 (%)			
Chemotherapy											
Ford 2014 [17]	84 84	Docetaxel + BSC BSC	69 (82) 67 (80)	65 (29–84) 66 (36–84)	73 (87) 74 (88)	0–2	70 (83) 72 (86)	14 (17) 12 (14)	2nd	Fluoropyrimidine + platinum	OS
Thuss-Patience 2011 [18]	21 19	Irinotecan BSC	18 (86) 11 (58)	58 (43–73) 55 (35–72)	21 (100) 19 (100)	0–2	17 (81) 14 (74)	4 (19) 5 (26)	2nd	Fluoropyrimidine + platinum or taxane	OS
Kang 2012 [19]	133 69	Docetaxel or irinotecan BSC	93 (70) 44 (64)	56 (31–83) 56 (32–74)	133 (100) 69 (100)	0–1	133 (100) 69 (100)	0 (0) 0 (0)	2nd or 3rd	Fluoropyrimidine + platinum	OS
Hironaka 2013 [20]	108 111	Paclitaxel Irinotecan	84 (78) 87 (78)	65 (37–75) 65 (38–75)	108 (100) 11 (100)	0–2	104 (96) 107 (96)	4 (4) 4 (4)	2nd	Fluoropyrimidine + platinum	OS
Nishikawa 2015a [21]	43 42 20 22	Paclitaxel Irinotecan Paclitaxel + S-1 Irinotecan + S-1	35 (81) 30 (71) 12 (60) 15 (68)	65 (31–74) 65 (44–74) 63 (37–74) 67 (47–73)	NA NA NA NA	0–2	41 (95) 42 (100) 20 (100) 21 (95)	2 (5) 0 (0) 0 (0) 2 (5)	2nd	Fluoropyrimidine + platinum	OS
Roy 2013 [22]	44 44 44	Docetaxel Irinotecan PEP-02	34 (77) 34 (77) 35 (79)	58 (33–81) 62 (33–79) 56 (38–81)	43 (98) 40 (91) 43 (98)	0–2	40 (91) 41 (93) 41 (93)	4 (9) 3 (7) 3 (7)	2nd	Not specified	ORR
Higuchi 2014 [23]	64 63	Irinotecan + cisplatin Irinotecan	49 (77) 55 (87)	66 (29–80) 67 (49–78)	44 (69) 40 (63)	0–2	64 (100) 63 (100)	0 (0) 0 (0)	2nd	Fluoropyrimidine + platinum or taxane	PFS
Nishikawa 2015b [24]	84 84	Irinotecan + cisplatin Irinotecan	68 (81) 63 (75)	67 (36–85) 68 (35–87)	64 (78) 71 (84)	0–1	84 (100) 84 (100)	0 (0) 0 (0)	2nd	Fluoropyrimidine monotherapy	OS
Kim 2015a [25]	23 25 23	Docetaxel + cisplatin Docetaxel + S-1 Docetaxel	21 (87) 15 (60) 18 (78)	55 (38–74) 55 (39–68) 56 (34–68)	23 (100) 25 (100) 23 (100)	0–2	22 (92) 23 (92) 23 (100)	2 (8) 2 (8) 0 (0)	2nd	Fluoropyrimidine + cisplatin	ORR
Kim 2015b [26]	25 27	Docetaxel + oxaliplatin Docetaxel	18 (72) 24 (89)	59 54	NA NA	0–2	24 (96) 26 (96)	1 (4) 1 (4)	2nd	Fluoropyrimidine + cisplatin	ORR
Nakanishi 2015 [27]	38 40	Paclitaxel + S-1 Paclitaxel	29 (76) 34 (85)	64 (42–79) 62 (38–80)	NA NA	0–2	37 (97) 46 (93)	1 (3) 3 (7)	2nd	Fluoropyrimidine + platinum	PFS
Tanabe 2015 [28]	145 148	Irinotecan + S-1 Irinotecan	99 (68) 109 (74)	67 (37–84) 66 (22–83)	NA NA	0–1	145 (100) 148 (100)	0 (0) 0 (0)	2nd	Fluoropyrimidine-based regimen	OS
Sym 2013[29]	30 29	Irinotecan + 5-FU/Lv Irinotecan	14 (47) 20 (69)	61 (30–75) 60 (45–76)	28 (93) 27 (93)	0–2	27 (90) 27 (93)	3 (10) 2 (7)	2nd	Fluoropyrimidine + platinum	ORR
Maruta 2007 [30]	12 12	Docetaxel + 5′DFUR Docetaxel	9 (75) 9 (75)	61 (38–74) 65 (59–71)	NA NA	0–2	11 (92) 11 (92)	1 (8) 1 (8)	2nd	Fluoropyrimidine + platinum	ORR
Nishina 2015 [31]	49 51	5-FU + methotrexate Paclitaxel	33 (67) 36 (71)	59 (30–74) 64 (39–75)	49 (100) 51 (100)	0–2	48 (98) 49 (96)	1 (2) 2 (4)	2nd	Fluoropyrimidine + cisplatin or methotrexate	OS
Targeted therapy											
Fuchs 2014 [5]	238 117	Ramucirumab BSC	169 (71) 79 (68)	60 (52–67) 60 (51–71)	NA NA	0–1	238 (100) 116 (99)	0 (0) 1 (1)	2nd	Fluoropyrimidine + platinum	OS
Ohtsu 2013 [12]	439 217	Everolimus Placebo + BSC	322 (73) 161 (74)	62 (20–86) 62 (20–88)	439 (100) 217 (100)	0–2	413 (94) 190 (87)	25 (6) 27 (12)	2nd or 3rd	Fluoropyrimidine + platinum	OS
Pavlakis 2015 [32]	97 50	Regorafenib BSC	78 (80) 40 (80)	NA NA	NA NA	0-1	97 (100) 50 (100)	0 (0) 0 (0)	2nd or 3rd	Fluoropyrimidine + platinum	PFS
Wilke 2014 [6]	330 335	Ramucirumab + paclitaxel Paclitaxel + placebo	229 (69) 243 (73)	61 (25–83) 61 (24–84)	NA NA	0–1	330 (100) 335 (100)	0 (0) 0 (0)	2nd	Fluoropyrimidine + platinum	OS
Bang 2015a [33]	62 62	Olaparib + paclitaxel Paclitaxel	49 (79) 44 (71)	63 (31–77) 61 (26–79)	NA NA	0-2	62 (100) 60 (96.8)	0 (0) 2 (3)	2nd	Fluoropyrimidine + platinum	PFS
Yi 2012 [11]	56 49	Sunitinib + docetaxel Docetaxel	40 (71) 33 (67)	54 (20–72) 52 (36–70)	47 (84) 47 (96)	0–2	30 (89) 46 (94)	6 (11) 3 (6)	2nd or 3rd	Fluoropyrimidine + platinum	TTP
Moehler 2013 [34]	45 46	Sunitinib + irinotecan + 5-FU/Lv Irinotecan + 5-FU/Lv	NA NA	NA NA	NA NA	KPS 100–70 %	NA NA	NA NA	2nd or 3rd	Taxane and/or platinum	PFS
Satoh 2015 [10]	40 42	Nimotuzumab + irinotecan Irinotecan	33 (82) 33 (79)	60 (27–75) 63 (32–75)	39 (97.5) 42 (100)	0–1	40 (100) 42 (100)	0 (0) 0 (0)	2nd or 3rd	Fluoropyrimidine-based regimen	PFS
Bang 2015b [35]	41 30	AZD-4547 Paclitaxel	29 (71) 22 (73)	63 62	NA NA	NA	NA NA	NA NA	2nd or 3rd	Not specified	PFS
Satoh 2014 [36]	132 129	Lapatinib + paclitaxel Paclitaxel	101 (77) 106 (82)	61 (32–79) 62 (22–80)	127 (96) 121 (94)	0–1	132 (100) 129 (100)	0 (0) 0 (0)	2nd	Fluoropyrimidine + cisplatin	OS
Lorenzen 2015 [37]	18 19	Lapatinib + capecitabine Lapatinib	17 (94) 14 (74)	56 (44–75) 62 (46–76)	18 (100) 19 (100)	0–2	16 (88) 18 (95)	2 (11) 1 (5)	2nd	Fluoropyrimidine + platinum	ORR
Li 2013 [13]	47 46 48	Apatinib 850 mg once daily Apatinib 425 mg twice daily Placebo	39 (83) 34 (74) 36 (75)	55 53 54	43 (91) 45 (98) 48 (100)	0–1	47 (100) 46 (100) 48 (100)	0 (0) 0 (0) 0 (0)	3rd or later	Fluoropyrimidine + platinum	PFS
Li 2016 [14]	176 91	Apatinib 850 mg once daily Placebo + BSC	132 (75) 69 (76)	58 (32–71) 58 (28–70)	NA NA	0–1	176 (100) 91 (100)	0 (0) 0 (0)	3rd or later	Fluoropyrimidine + platinum	OS

The most important baseline characteristics of all 28 studies are shown

*5-FU* 5-fluorouracil, *BSC* best supportive care, *ECOG PS* Eastern Collaborative Oncology Group performance status, *GEJ* gastro-esophageal junction, *KPS* Karnofsky performance status, *Lv* leucovorin, *NA* not available, *NR* not reached

### Single cytotoxic agent compared to best supportive care

**Fig. 3:**
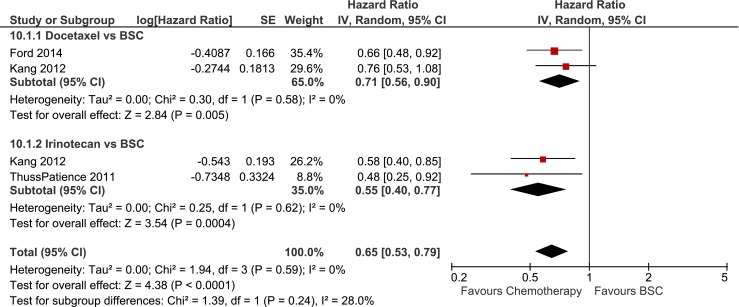
Overall survival in studies comparing single-agent taxane and irinotecan to best supportive care. Forest-plot of single-agent taxane and irinotecan compared to best supportive care in terms of overall survival (A). *BSC* best supportive care, *IRI* irinotecan, *TAX* taxane, *PTX* paclitaxel, *DTX* docetaxel

Increased overall survival was found for single cytotoxic agents *vs*. BSC (HR 0.65, 0.53–0.79) by meta-analysis of 3 studies including 410 patients as shown in Fig. 3 [17–19]. In subgroup analysis, increased OS was shown for both taxane (HR 0.71, 0.56–0.90) and irinotecan (HR 0.55, 0.40–0.77) compared to BSC. Absolute median survival gain ranged from Δ1.4 to Δ1.6 months for taxane compared to BSC and ranged from Δ1.6 to Δ2.7 months for irinotecan compared to BSC (Table 3). Both taxane and irinotecan were associated with statistically significant increased grade 3–4 neutropenia (33/207 *vs*. 2/198, RR 12.17, 3.41–43.50) and febrile neutropenia (9/100 *vs*. 0/91, RR 8.69, 1.14–66.42) compared to BSC.

**Table 3:** Efficacy of second-line chemotherapy

Study	Efficacy sample	Arms	Overall survival				Progression-free survival			
			Median	Median difference	HR (95 % CI)	*P*	Median	Median difference	HR (95 % CI)	*P*
Taxane/irinotecan *vs*. BSC										
Ford 2014 [17]	84 84	Docetaxel BSC	5.2 3.6	Δ 1.6	0.66 (0.48–0.92)	*0.01	NA NA	NA	NA	NA
Thuss-Patience 2011 [18]	21 19	Irinotecan BSC	4.0 2.4	Δ 1.6	0.48 (0.25–0.92)	*0.02	NA NA	NA	NA	NA
Kang 2012 [19]	66 60 69	Docetaxel Irinotecan BSC	5.2 6.5 3.8	Δ 1.4 Δ 2.7	0.76 (0.53–1.08) 0.58 (0.40–0.85)	0.13 *0.01	NA NA NA	NA NA	NA NA	NA NA
Taxane-based *vs*. irinotecan-based regimens										
Kang 2012 [19]	66 60	Docetaxel Irinotecan	5.2 6.5	Δ −1.3	1.31 (0.78–2.20)	0.12	NA NA	NA	NA	NA
Hironaka 2013 [20]	108 111	Paclitaxel Irinotecan	9.5 8.4	Δ 1.1	0.88 (0.67–1.16)	0.38	3.6 2.3	Δ 1.3	0.87 (0.67–1.14)	0.33
Nishikawa 2015a [21]	63 64	S-1 + paclitaxel and paclitaxel-alone S-1 + irinotecan and irinotecan-alone	11.1 11.8	Δ −0.7	0.98 (0.68–1.42)	0.92	4.1 3.6	Δ 0.5	0.67 (0.47–0.97)	*0.03
Roy 2013 [22]	44 44 44	Docetaxel Irinotecan PEP02	7.7 7.8 7.3	Δ 0.2	0.83 (0.54–1.27)	0.51	2.7 2.6 2.7	Δ 0.1	1.00 (0.68–1.47)	0.38
Combination therapy *vs*. taxane/irinotecan-alone										
Cisplatin-based										
Nishikawa 2015b [24]	84 84	Cisplatin + irinotecan Irinotecan	13.9 12.7	Δ 1.2	0.83 (0.60–1.17)	0.29	2.6 2.1	Δ 0.5	0.86 (0.61–1.20)	0.38
Higuchi 2014 [23]	64 63	Cisplatin + irinotecan Irinotecan	10.7 10.1	Δ 0.6	1.00 (0.69–1.44)	0.98	3.8 2.8	Δ 1.0	0.68 (0.47–0.98)	*0.04
Kim 2015a [25]	23 23	Cisplatin + docetaxel Docetaxel	5.6 10.0	Δ −4.4	1.34 (1.02–1.77)	*0.03	1.8 1.3	Δ 0.5	0.96 (0.72–1.29)	0.80
Oxaliplatin-based										
Kim 2015b [26]	25 27	Oxaliplatin + docetaxel Docetaxel	8.1 7.2	Δ 0.9	0.87 (0.65–1.16)	0.35	4.9 2.0	Δ 2.9	0.64 (0.48–0.85)	*<0.01
Fluoropyrimidine-based										
Nishikawa 2015a [21]	42 85	S-1 + paxlitaxel and S-1 + irinotecan Paclitaxel-alone and irinotecan-alone	11.3 11.1	Δ 0.2	0.95 (0.64–1.41)	0.81	3.7 3.7	Δ 0.0	1.01 (0.69–1.49)	0.93
Kim 2015a [25]	25 23	S-1 + docetaxel Docetaxel	6.9 10.0	Δ 3.1	1.12 (0.84–1.50)	0.42	2.7 1.3	Δ 1.4	0.73 (0.54–0.98)	*0.03
Nakanishi 2015 [27]	38 40	S-1 + paclitaxel Paclitaxel	10.0 10.0	Δ 0.0	0.83 (0.51–1.36)	NA	4.6 4.6	Δ 0.0	0.86 (0.54–1.37)	NA
Tanabe 2015 [28]	145 148	S-1 + irinotecan Irinotecan	8.8 9.5	Δ −0.7	0.99 (0.78–1.25)	0.92	3.8 3.4	Δ 0.4	0.85 (0.67–1.07)	*0.02
Sym 2013[29]	30 29	5-FU/Lv + irinotecan Irinotecan	6.7 5.8	Δ 0.9	0.83 (0.47–1.45)	0.51	3.0 2.2	Δ 0.8	0.83 (0.50–1.39)	0.48
Maruta 2007 [30]	12 12	5′DFUR + docetaxel Docetaxel	7.6 4.0	Δ 3.6	NA	*<0.05	NA NA	NA	NA	NA
5-FU + methotrexate										
Nishina 2015 [31]	49 51	5-FU + methotrexate Paclitaxel	7.7 7.7	Δ 0.0	1.13 (0.73–1.75)	0.30	2.4 3.7	Δ -1.3	1.76 (1.15–2.70)	*<0.01

To summarize, treatment efficacy, median overall survival (months), median progression-free survival, hazard ratios (HR), 95 % confidence intervals (95 % CI), and *p* values are shown for all chemotherapy studies

Notes: *P < 0.05

*5-FU* 5-fluorouracil, *95 % CI* 95 % confidence interval, *BSC* best supportive care, *RR* risk ratio, *Lv* leucovorin, *NA* not available

Taxane or irinotecan as second-line monotherapy can be used in the second-line setting to treat patients with a performance status of 0 to 2, but the modest absolute survival benefit compared to best supportive care should be considered.

### Taxane-based compared to irinotecan-based chemotherapy

**Fig. 4:**
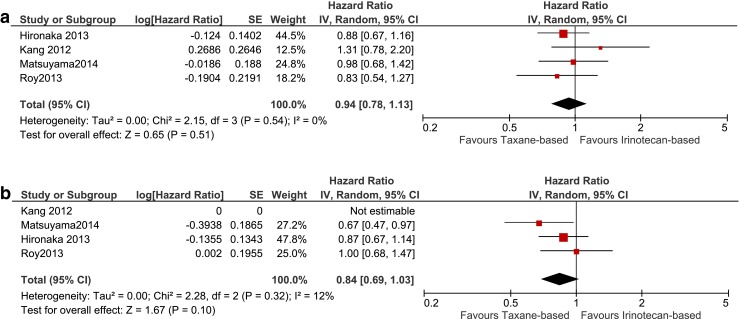
Studies comparing taxane-based and irinotecan-based chemotherapy. Forest-plot of taxane-based compared to irinotecan-based chemotherapy terms of overall survival (**a**) and progression-free survival (**b**). Notes: Roy 2012 irinotecan and PEP02 arms were pooled and compared to the docetaxel arm. *BSC* best supportive care, *IRI* irinotecan, *TAX* taxane, *PTX* paclitaxel, *DTX* docetaxel

Meta-analysis of four studies including 604 patients showed that there was no difference between taxane-based and irinotecan-based regimens in OS (HR 0.94, 0.78–1.13) and PFS (HR 0.84, 0.69–1.03), with absolute median OS gains ranging from Δ−1.3 to Δ1.1 months and absolute PFS gains ranging from Δ0.1 to Δ1.3 months (Fig. 4; Table 3)[19–22]. Irinotecan was associated with increased grade 3–4 neutropenia, diarrhea and anorexia compared to taxane, whereas taxane was associated with increased neuropathy (Table 3). In sum, taxane and irinotecan were similar in efficacy. For an individual patient, a taxane or irinotecan can be chosen based on the specific toxicity profile of these agents. Taxane and irinotecan will be regarded as comparators in the next sections.

### Combination chemotherapy compared to single-agent taxane or irinotecan

**Fig. 5:**
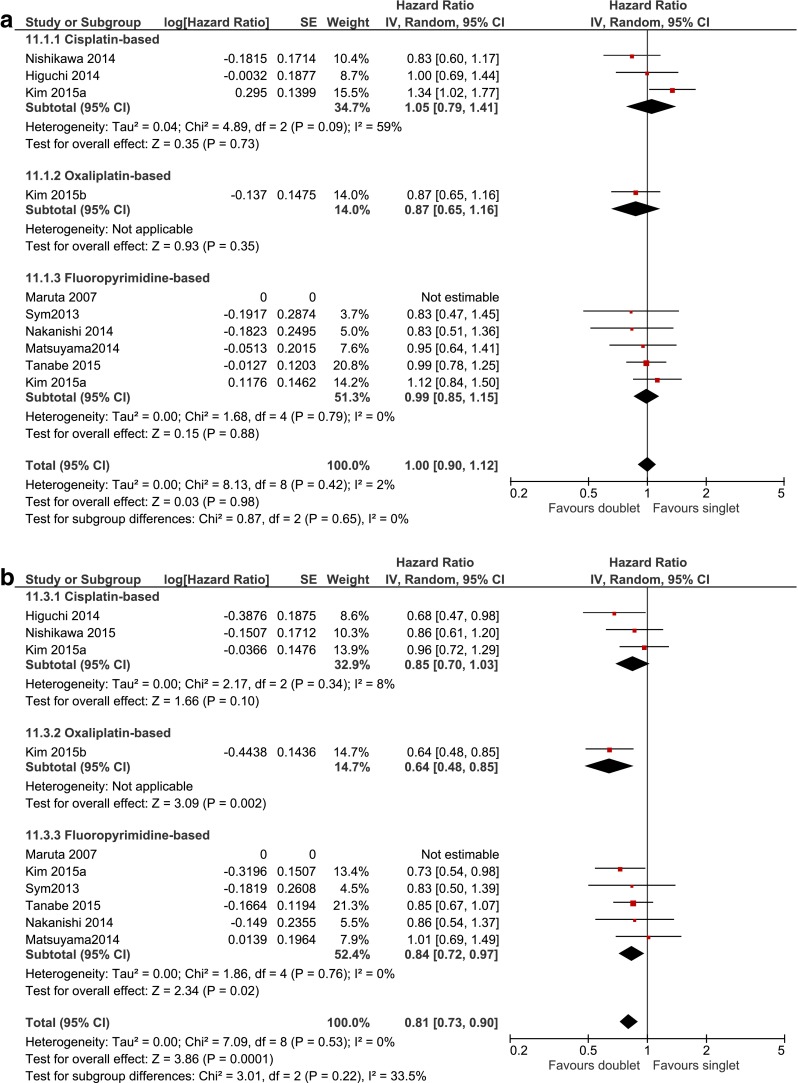
Studies comparing doublet and single-agent chemotherapy. The efficacy of doublet chemotherapy regimen, consisting of a taxane or irinotecan backbone combined with cisplatin, oxaliplatin, or fluoropyrimidine, *vs*. taxane or irinotecan single agent in terms of overall survival (**a**) and progression-free survival (**b**). *BSC* best supportive care, *IRI* irinotecan, *TAX* taxane, *PTX* paclitaxel, *DTX* docetaxel

The effect of adding cisplatin, oxaliplatin or fluoropyrimidine to single-agent irinotecan or taxane was assessed in three studies including 341 patients [23–25], in one study including 52 patients [26] and in six studies including 629 patients [21, 25, 27–30] respectively (Fig. 5; Table 3). A HR for OS and PFS could not be calculated for one small study [30].

Meta-analysis showed that doublets were not more effective compared to single agents in OS (HR 1.00, 0.90–1.12) and no significant differences were found with subgroup analysis by type of additional cytotoxic agent. On the other hand, the pooled effect for PFS was significant (HR 0.81, 0.73–0.90). Subgroup analysis showed that the addition of oxaliplatin to the taxane or irinotecan backbone was associated with increased PFS with HR 0.64 (0.48–0.85) and absolute median PFS gain of Δ2.9 months. Also, the addition of fluoropyrimidine resulted in longer PFS with HR 0.84 (0.72-0.97), but absolute median PFS gain ranged from Δ0 to Δ1.4 months only. The cisplatin-based subgroup did not reach statistical significance over monotherapy. No statistically significant heterogeneity was detected in any of the analyses (Fig. 5; Table 3). Overall, none of the grade 3–4 adverse events showed statistically significant differences between doublet and monotherapy, although a general trend towards increased toxicity could be observed for doublets (Table 4). Exploratory subgroup analysis showed that oxaliplatin-based doublets were associated with significantly increased grade 3–4 neutropenia (8/25 *vs*. 0/27, RR 18.31, 1.11–301.60) and fluoropyrimidine-based doublets with increased grade 3–4 anemia (37/292 *vs*. 26/336, RR 1.65, 1.02–2.66).

**Table 4:** Grade 3–4 adverse events of second-line chemotherapy

Grade 3–4 AE	Irinotecan-based *vs*. taxane-based chemotherapy									Combination chemotherapy *vs*. chemotherapy-alone								
	Irinotecan		Taxane		Estimate			Heterogeneity		Doublet		Singlet		Estimate			Heterogeneity	
	*n*	*N*	*n*	*N*	RR (95 % CI)	*P*	Trials	*I* ^2^ (%)	*P*	*n*	*N*	*n*	*N*	RR (95 % CI)	*P*	Trials	*I* ^2^ (%)	*P*
Hematological																		
Neutropenia	82	321	55	282	1.40 (1.04–1.88)	*0.03	4	0	0.74	144	487	124	533	1.21 (0.98–1.49)	0.07	10	0	0.58
Leukopenia	27	173	25	172	1.07 (0.60–1.91)	0.81	2	10	0.29	59	398	50	440	0.91 (0.47–1.76)	0.79	8	56	*0.03
Trombocytopenia	8	321	4	282	1.65 (0.51–5.30)	0.40	4	0	0.94	8	408	8	454	1.16 (0.44–3.04)	0.76	7	0	0.72
Anemia	62	321	53	282	1.16 (0.84–1.60)	0.36	4	0	0.64	63	462	41	506	1.61 (0.97–2.66)	0.06	9	22	0.24
Febrile neutropenia	18	261	10	216	1.30 (0.25–6.78)	0.75	3	63	0.07	24	393	13	440	1.68 (0.52–5.42)	0.39	8	42	0.10
Non-hematological																		
Diarrhea	5	110	1	282	5.06 (1.85–13.87)	*0.002	4	0	0.74	16	475	21	521	0.89 (0.47–1.70)	0.73	9	0	0.77
Nausea	19	321	7	282	2.02 (0.82–4.99)	0.13	4	0	0.51	21	434	23	481	1.01 (0.57–1.79)	0.98	8	0	0.88
Vomiting	11	261	7	216	1.09 (0.40–2.97)	0.87	3	0	0.48	5	334	10	380	0.65 (0.22–1.90)	0.43	5	0	0.79
Fatigue	13	211	20	174	0.82 (0.25–2.66)	0.74	3	46	0.16	22	392	19	439	1.29 (0.70–2.39)	0.42	7	0	0.66
Anorexia	35	321	15	282	2.06 (1.13–3.73)	*0.02	4	0	0.51	58	462	57	506	1.11 (0.78–1.56)	0.56	9	0	0.80
Stomatitis	3	60	2	66	1.65 (0.29–9.54)	0.58	1	NA	NA	4	59	3	58	1.28 (0.29–5.69)	0.75	3	0	0.51
Neuropathy	0	110	8	108	0.06 (0.00–0.99)	*0.05	1	NA	NA	2	64	2	61	0.91 (0.14–6.00)	0.92	2	0	0.37
Toxicity-related death	NA	NA	NA	NA	NA	NA	NA	NA	NA	0	175	3	178	0.25 (0.03–2.27)	0.22	2	0	0.84

Grade 3–4 adverse events of taxane-based *vs*. irinotecan-based chemotherapy (*left*) and doublet *vs*. single-agent chemotherapy (*right*)

Notes: the irinotecan and PEP02 arms from Roy 2012 were pooled and compared to the docetaxel arm

*P < 0.05

*5-FU* 5-fluorouracil, *95 % CI* 95 % confidence interval, *Lv* leucovorin, *RR* risk ratio, *NA* not available

One study including 100 patients was analyzed separately, since only patients with peritoneal metastasis were included and seven out of 48 patients (15 %) in the methotrexate plus 5-FU combination arm did in fact receive 5-FU monotherapy [31]. Taxane monotherapy was associated with increased PFS (HR 0.57, 0.37–0.88) compared to a combination of methotrexate and 5-FU or 5-FU monotherapy (Table 3). There was no difference in OS. An increased rate of grade 3–4 neutropenia was found for methotrexate plus 5-FU *vs*. taxane (14/49 *vs*. 6/51, RR 2.43, 1.02–5.81). In sum, combination chemotherapy is not recommended as second-line treatment due to lack of superior efficacy at the cost of additional toxicity.

### Single targeted agents compared to best supportive care

Meta-analysis was only possible for apatinib compared to placebo with 2 studies including 408 patients [13, 14], since all other targeted agents were investigated in one study only. In Table 5, the efficacy and statistically significant grade 3–4 adverse events of single targeted agents compared to BSC and single cytotoxic agents are summarized.

**Table 5:** Efficacy and safety of second- or third-line targeted therapy

Study	Efficacy sample size	Arms	Treatment line	Overall survival				Progression-free survival				Safety		
				Median	Median difference	HR (95%CI)	*P*	Median	Median difference	HR (95%CI)	*P*	Safety sample size	Grade 3–4 Toxicity	Exp *n* (%) *vs*. control *n* (%)
Single targeted agent														
Ramucirumab			2nd											
Fuchs 2014 [5]	238 117	Ramucirumab BSC		5.2 3.8	Δ1.4	0.78 (0.60–1.00)	*0.05	2.1 1.3	Δ 0.8	0.48 (0.38–0.62)	*<0.01	236 115	No differences	
Everolimus			2nd or 3rd											
Ohtsu 2013 [12]	439 217	Everolimus Placebo		5.4 4.3	Δ 1.1	0.90 (0.75–1.08)	0.12	1.7 1.4	Δ 0.3	0.66 (0.56–0.78)	*<0.01	437 215	Anorexia Hypokalemia Thrombocytopenia Stomatitis	48 (11 %) *vs*. 12 (6 %) 26 (6 %) *vs*. 2 (1 %) 22 (5 %) *vs*. 3 (1 %) 20 (5 %) *vs*. 0 (0 %)
Regorafenib			2nd or 3rd											
Pavlakis 2015 [32]	97 50	Regorafenib Placebo		5.8 4.5	Δ 1.3	0.74 (0.51–1.08)	0.11	2.5 0.9	Δ 1.6	0.41 (0.28–0.59)	*<0.01	97 50	No differences	
Targeted agent + chemotherapy														
Ramucirumab + taxane			2nd											
Wilke 2014 [6]	330 335	Ramucirumab + Paclitaxel Paclitaxel + placebo		9.6 7.4	Δ 2.2	0.81 (0.68–0.96)	*0.02	4.4 2.9	Δ 1.5	0.64 (0.54–0.75)	*<0.01	327 329	Hypertension Fatigue Neuropathy	48(15 %) *vs*. 19 (6 %) 39 (12 %) *vs*. 18 (5 %) 27 (8 %) *vs*. 15 (5 %)
Sunitinib + chemotherapy			2nd or 3rd											
Yi 2012 [11]	56 49	Sunitinib + docetaxel Docetaxel		8.0 6.6	Δ 1.4	0.94 (0.60–1.49)	0.80	NA	NA	0.77 (0.52–1.16)	0.21	56 49		
Moehler 2013 [34]	45 46	Sunitinib + Irinotecan + 5-FU/Lv Irinotecan + 5-FU/Lv		10.5 9.0	Δ 1.5	0.82 (0.50–1.34)	0.42	3.6 3.3	Δ 0.3	1.10 (0.70–1.74)	0.66	45 46		
Pooled		Sunitinib + CT *vs*. CT				0.88 (0.63–1.24)	0.47			0.91 (0.65–1.28)	0.59		Neutropenia	43 (43 %) *vs*. 19 (20 %)
Nimotuzumab + Irinotecan			2nd or 3rd											
Satoh 2015 [10]	40 42	Nimotuzumab + Irinotecan Irinotecan		8.2 7.6	Δ 0.6	0.99 (0.62–1.60)	0.98	2.4 2.8	Δ -0.4	0.86 (0.52–1.44)	0.57	40 42	No differences	
Olaparib + taxane			2nd											
Bang 2015a [33]	62 62	Olaparib + paclitaxel Paclitaxel		13.1 8.3	Δ 4.8	0.56 (0.35–0.87)	*0.01	3.9 3.5	Δ 0.4	0.80 (0.54–1.18)	0.13	61 62	Neutropenia	34 (56 %) *vs*. 24 (39 %)
Targeted agents for specific molecular prespecified subgroups														
HER-2 positive			2nd											
Satoh 2014 [36]	132 129	Lapatinib + paclitaxel Paclitaxel		11.0 8.9	Δ 2.1	0.84 (0.64–1.11)	0.10	5.5 4.4	Δ 1.1	0.85 (0.63–1.13)	0.15	131 129	Febrile neutropenia Diarrhea	9 (7 %) *vs*. 2 (2 %) 23 (18 %) *vs*. 0 (0 %)
Lorenzen 2015 [37]	18 19	Capecitabine + lapatinib Lapatinib		NR 4.7	NC	1.06 (0.34–3.29)	0.92	1.5 1.3	Δ 0.2	NA	NS	18 19	No differences	
Fibroblast growth factor receptor 2 amplification			2nd or 3rd											
Bang 2015b [35]	41 30	AZD-4547 Paclitaxel		NA NA	NA	NA NA		1.8 3.5	Δ 1.7	1.57 (1.12–2.21)	NS		Not reported	
Third- or further-line single targeted agent														
Apatinib			3rd or further											
Li 2016 [14]	176 91	Apatinib Placebo		6.5 4.7	Δ 1.8	0.71 (0.54–0.94)	*0.01	2.6 1.8	Δ 0.8	0.44 (0.33–0.60)	*<0.01	176 91		
Li 2013 [13]	47 46 48	Apatinib 850 mg Apatinib 425 mg Placebo		4.8 4.3 2.5	Δ 2.3 Δ 1.8	0.37 (0.22–0.62) 0.41 (0.24–0.71)	*<0.01 *<0.01	3.7 3.2 1.4	Δ 2.3 Δ 1.8	0.18 (0.10–0.34) 0.21 (0.11–0.38)	*<0.01 *<0.01	47 46 48		
Pooled		Apatinib *vs*. placebo				0.50 (0.32–0.79)	*<0.01			0.27 (0.14–0.51)	*<0.01		HFS Hypertension	23 (9 %) *vs*. 1 (%) 17 (6 %) *vs*. 0 (0 %)

To summarize treatment efficacy of targeted therapy, median overall survival (months), median progression-free survival and hazard ratios (HR) with 95 % confidence intervals (95 % CI) were shown. For safety of targeted therapy, only grade 3–4 AEs were reported for which a statistically significant difference exist between the occurrence in the treatments arms.

Notes: since more than one study was available for the comparisons for sunitinib and apatinib, also the pooled HRs were given.

*P < 0.05

*5-FU* 5-fluorouracil, *95 % CI* 95 % confidence interval, *BSC* best supportive care, *CT* chemotherapy, *d* days, *exp* experimental agent, *Lv* leucovorin, *HFS* hand-foot syndrome, *HR*: hazard ratio, *RR* risk ratio, *NA*: not available, *NC* not calculable, *NS*: not statistically significant

In second-line setting, ramucirumab monotherapy showed increased benefit in both OS, HR 0.78 (0.61–1.00) with absolute median OS gain of Δ1.4 months and in PFS, HR 0.48 (0.38–0.62) with absolute median PFS gain of Δ0.8 months compared to BSC. In second- or third-line setting, no OS benefit of the mammalian target of rapamycin (mTOR) inhibitor everolimus and the multityrosine kinase inhibitor regorafenib was found over BSC. Increased PFS was found for both everolimus, HR 0.66 (0.56–0.78), with median PFS gain of Δ0.3 months and for regorafenib, HR 0.41 (0.28–0.59), with median PFS gain of Δ1.6 months respectively. As third- or later-line therapy, apatinib, a tyrosine kinase inhibitor that selectively inhibits VEGFR-2, showed increased OS and PFS, HR 0.50 (0.32–0.79) and HR 0.27 (0.14–0.51) *vs*. BSC, with a median OS gain ranging from Δ1.8 to Δ2.3 months and PFS gain ranging from Δ0.8 to Δ2.3 months.

In Table 5, only grade 3–4 AEs were reported for which a statistically significant difference exist between the occurrence in the treatments arms. Compared to BSC, significantly increased grade 3–4 toxicities were anorexia, hypokalemia, thrombocytopenia, and stomatitis for second-or third-line everolimus, and hand-foot syndrome and hypertension with third- or later-line apatinib (Table 5). None of the AEs associated with second-line ramucirumab and second- or third-line regorafenib reached statistical significance compared to BSC. In sum, in third-line single-agent apatinib and in second-line single-agent ramucirumab may be considered for patients with performance status 0 or 1 who cannot or do not want to undergo chemotherapy. However, the modest absolute survival benefit compared to best supportive care should be taken into consideration.

### The addition of a targeted agent to chemotherapy compared to chemotherapy-alone

In second-line setting, increased OS was shown for ramucirumab plus taxane (HR 0.81 0.68–0.96), with a median survival gain of Δ2.2 months, and for the enzyme poly-ADP ribose polymerase [PARP] inhibitor olaparib plus taxane (HR 0.56, 0.35–0.87), with a median survival gain of Δ4.8 months compared to taxane-alone. Also, increased PFS was found for ramucirumab plus taxane (HR 0.64, 0.54–0.75), with a median PFS gain of Δ1.5 months, but not for olaparib plus taxane (Table 5). In second- or third-line setting, the epidermal growth factor receptor [EGFR] inhibitor nimotuzumab plus irinotecan and the multityrosine kinase inhibitor sunitinib plus irinotecan-based chemotherapy did not show any significant difference in OS and PFS compared to chemotherapy-alone (Table 5). Compared to chemotherapy-alone, second-line ramucirumab plus taxane was associated with increased grade 3–4 hypertension, fatigue and neuropathy and both second-line olaparib plus taxane and second-or third-line sunitinib plus chemotherapy were associated with increased neutropenia (Table 5). None of the AEs associated with second- or third-line nimotuzumab plus taxane reached statistical significance compared to taxane-alone.

In sum, based on results of phase III studies ramucirumab plus taxane is the only combination therapy that can be recommended as second-line therapy for patients with a performance status of 0–1. Olaparib in combination with a taxane shows potential as a second-line regimen when results are confirmed by phase III results.

### Targeted agents in specific molecular sub-populations

In HER-2 positive patients, the addition of lapatinib, a dual inhibitor of EGFR and HER-2 tyrosine kinase activity, to a taxane as second-line regimen was not associated with increased efficacy in OS and PFS over taxane-alone (Table 5). However, a significant effect was observed in the immunohistochemistry (IHC) 3+ subgroup for OS (HR 0.59, 0.37-0.93) and PFS (HR 0.54 0.33-0.90) [36]. The addition of capecitabine to lapatinib *vs*. lapatinib alone showed no difference in both OS and PFS in an HER-2 positive population [37]. Furthermore, one small study failed to demonstrate a benefit in OS or PFS for the fibroblast growth factor receptor (FGFR)1-3 inhibitor AZD-4547 over taxane-alone in a FGFR-2-amplificated population [35] (Table 5). In sum, there is no evidence for HER-2 directed second-line therapy, although lapatinib plus taxane showed promising efficacy in patients with HER2 IHC 3+.

### Best supportive care: palliative treatment for obstruction and dysphagia

Stent placement, intraluminal brachytherapy, or intraluminal balloon dilatation are widely accepted palliative procedures to relief obstruction and dysphagia caused by locally advanced esophageal tumours [38, 39]. On the one hand, stent placement can provide rapid palliation but there is a risk of complications, compared to brachytherapy. On the other hand, brachytherapy is associated with long-term relief and with fewer complications compared to stent placement, but it takes longer before relief of symptoms is initiated [40]. Also, it has been shown that the combination of both brachytherapy and stent placement is more effective in survival and symptom relief compared to stent placement-alone [41]. Guidelines recommend the concurrent use of these palliative procedures and multimodality therapy, for example chemotherapy or targeted therapy, but these procedures should be carefully chosen based on the patients’ prognosis and needs.

## Discussion

In this systematic review we showed that both taxane and irinotecan as single agents significantly prolonged survival compared to BSC. Although the hazard ratios were statistically significant, the absolute survival benefit was marginal and this should be taken into consideration in clinical practice. In contrast to earlier meta-analyses, the current meta-analysis provided evidence that taxane and irinotecan-based regimens are equally effective in terms of both OS and PFS. However, the two regimens showed a different toxicity profile, which may guide clinical decision-making in the use of a specific cytotoxic agent in an individual patient.

No OS benefit was detected for the addition of another cytotoxic agent (i.e., platinum or fluoropyrimidine) to a backbone of taxane or irinotecan. The addition of fluoropyrimidine significantly resulted in a statistically significant pooled HR of 0.84, but it is debatable whether a 16 % risk reduction of PFS is clinically relevant. According to a large international expert consensus panel, OS as endpoint in oncology clinical trials is more appropriate and a HR ≤ 0.80 is clinically relevant [15]. The panel stated a similar or even stricter criterion for PFS compared to the criterion of OS. The HR of 0.84 in the current meta-analysis does not meet the criterion of HR ≤ 0.80 for PFS. Moreover, the majority of the studies within this comparison did not show any absolute gain in median PFS. Of note, PFS was prolonged by the addition of oxaliplatin (HR 0.64, 0.48–0.85) in a small phase II study with patients that received cisplatin in the first-line treatment [26], so the potential of oxaliplatin for cisplatin-refractory patients could be subject of a larger prospective study. Based on this evidence, and acknowledging that toxicity was increased with combination therapy, we conclude that there is currently no role for combination chemotherapy in the second-line setting.

Regarding targeted therapy, the current systematic review provided evidence from phase III studies for three treatments. Second-line ramucirumab plus taxane significantly prolonged OS and PFS compared to taxane-alone with a clinically relevant absolute survival gain in patients with performance status 0 or 1 [6]. On the other hand, second-line ramucirumab as monotherapy showed a marginal absolute survival gain compared to BSC [5]. Apatinib monotherapy is currently the only treatment that has been tested in a third- or later-line setting (in both phase II and III studies) and might be clinically relevant in terms of relative and absolute gain in survival for patients with ECOG performance status 0 or 1 [13, 14]. In phase II studies, regorafenib monotherapy met its primary outcome criterion PFS by a HR of 0.41, but the median gain in PFS was only 1.6 months. Future large prospective studies should indicate if regorafenib could be utilized in second- or third-line setting [32]. Although olaparib plus taxane did not meet its primary endpoint PFS, OS was significantly prolonged as quantified by a HR of 0.56 and an absolute survival gain of median 4.8 months [34]. Results of a phase III RCT are awaited for olaparib (NCT01924533). Evidence for the addition of lapatinib to taxane in an HER-2 positive population was weak; only patients with HER2 IHC 3+ benefited from lapatinib while toxicity was increased [36].

We also discuss the limitations of this review. First, some studies with targeted agents, such as regorafenib monotherapy [32], were conducted in second- as well as in third-line setting. This makes the assessment of the specific indication for the targeted agent difficult. However, the study population is homogeneous as all included patients were refractory to fluoropyrimidine and platinum-based regimens. Furthermore, except for apatinib and sunitinib, meta-analysis was not possible for the majority of targeted agents since those were examined in a single study only. As most targeted agents have different mechanisms of action, pooling would introduce heterogeneity and would complicate the interpretation of results. Moreover, the overview of both relative and absolute efficacy results as well as the statistically significant grade 3–4 adverse events provided in Table 5 might be sufficient to value each targeted agent against current evidence.

In conclusion, based on the currently available phase III evidence, ramucirumab plus taxane can be regarded standard treatment for fit patients with a performance status of 0 or 1, who wish to undergo second-line treatment. The phase III results of olaparib in combination with paclitaxel are eagerly awaited. Combination chemotherapy has currently no role in the second-line treatment due to lack of efficacy. Taxane or irinotecan as monotherapy might be alternatives for patients with a performance status of 2 and thus not eligible for ramucirumab plus taxane or for patients with performance status 0 or 1 who prefer monotherapy. However, the modest absolute survival benefit of taxane or irinotecan monotherapy compared to best supportive care should be considered. Apatinib is a valuable option as third-line therapy for fit patients with a performance status of 0 or 1, again with limited absolute survival benefit. Finally, patient with a performance status larger than 2 after first- or second-line therapy, should be offered BSC.
